# Residual Right Coronary Artery Stenosis after Left Main Coronary Artery Intervention Increased the 30-Day Cardiovascular Death and 3-Year Right Coronary Artery Revascularization Rate

**DOI:** 10.1155/2020/4587414

**Published:** 2020-06-14

**Authors:** Chien-Ho Lee, Shaur-Zheng Chong, Shu-Kai Hsueh, Wen-Jung Chung, Cheng-I Cheng

**Affiliations:** Division of Cardiology, Department of Internal Medicine, Chang Gung Memorial Hospital, Kaohsiung, Taiwan

## Abstract

**Background:**

The outcomes of patients with concomitant left main coronary artery (LMCA) and right coronary artery (RCA) diseases are reportedly worse than those with only LMCA disease. To date, only few studies have investigated the clinical impact of percutaneous coronary intervention (PCI) on RCA stenosis during the same hospitalization, in which LMCA disease was treated. This study was aimed at comparing the outcomes between patients with and without right coronary artery intervention during the same hospital course for LMCA intervention.

**Methods and Results:**

From a total of 776 patients who were undergoing PCI to treat LMCA disease, 235 patients with concomitant RCA significant stenosis (more than 70% stenosis) were enrolled. The patients were divided into two groups: 174 patients received concomitant PCI for RCA stenosis during the same hospitalization, in which LMCA disease was treated, and 61 patients did not receive PCI for RCA stenosis. Patients without intervention to the right coronary artery had higher 30-day cardiovascular mortality rates and 3-year RCA revascularization rates compared to those with right coronary artery intervention. Patients without RCA intervention at the same hospitalization did not increase the 30-day total death, 3-year myocardial infarction rate, 3-year cardiovascular death, and 3-year total death.

**Conclusions:**

In patients with LM disease and concomitant above or equal to 70% RCA stenosis, PCI for RCA lesion during the same hospitalization is recommended to reduce the 30-day cardiovascular death and 3-year RCA revascularization rate.

## 1. Introduction

With recent significant advancement in devices and techniques used for percutaneous coronary intervention (PCI), unprotected left main (LM) coronary artery disease (CAD) can be safely and effectively managed. For patients with LM CAD who have a low-to-moderate SYNTAX score, the long-term results of PCI outcome are not inferior to coronary artery bypass graft (CABG), whereas coronary artery bypass graft (CABG) remains the primary choice for LM and multivessel CAD in those patients with a high SYNTAX score (>32) [1, 2]. However, PCI to left main disease is still the popular alternative treatment in the real world. In a recent meta-analysis, PCI to left main disease had the same all-cause mortality versus the CABG intervention [3]. However, the patients with concomitant LM and RCA disease had higher cardiac death after PCI to the left main disease than those without RCA disease (17.7% vs. 6.7%, *p*=0.056), and the patient with chronic total occlusion (CTO) of the right coronary artery had extreme high mortality (30% vs 6.7%, *p*=0.015) [4]. Residual CTO of the RCA is the predictor of mortality for patients who undergo PCI to unprotected left main disease [5, 6]. Another study also showed that patients with unprotected LM and concomitant RCA lesions who undergo PCI had worse early and long-term outcomes compared to those without RCA lesions [7]. Current ACC/AHA guideline recommends PCI to the noninfarct vessel in selected patients with STEMI, and ESC guideline recommends revascularization of the noninfarct-related artery in patients with ST-segment elevation myocardial infarction (STEMI) before hospital discharge [8, 9]. On the other hand, guidelines have not given any comments on the one-time revascularization in patients with multivessel CAD and left main disease. Till date, the impact of treating RCA disease during the same hospitalization for LM coronary artery disease treatment has not yet been well discussed.

There are limited data regarding the effects of PCI for RCA stenosis in patients who undergo PCI for unprotected LM CAD in the same hospitalization. Our previous study showed that PCI in these LM diseased patients in our hospital is feasible with a high success rate and is comparable to CABG in terms of cardiac death and overall survival, and these results are similar to most other studies [10–13]. This study was aimed at evaluating the impact of right coronary intervention at the same hospitalization for those patients with concomitant left main and right coronary artery stenosis and received left main PCI.

## 2. Methods

### 2.1. Study Population

Patients with unprotected left main disease and received PCI in our hospital between July 2001 and September 2016 were retrospectively enrolled in our study. Patients with acute ST-elevation myocardial infarction (STEMI), RCA stenosis less than 70%, or RCA with chronic total occlusion (CTO) were excluded in this study. The study protocol was approved by the Institutional Review Board of the Chang Gung Medical Foundation.

### 2.2. Study Design and Endpoints

Detailed informed consent for the PCI procedure was obtained from all patients before the procedure, and all treatment options were discussed with the patients and their families. All patients were treated with the dual antiplatelet therapy (aspirin plus one kind of P_2_Y_12_ inhibitor) after PCI procedure, and *β*-adrenergic blocker, angiotensin-converting enzyme inhibitor or angiotensin-receptor blocker, and statin were used as the standard therapy if patient had no contraindication.

These patients were divided into two groups, the group in which patients had RCA intervention at the same hospitalization (either in the same session or before discharge) was defined as the group of “with RCA intervention,” and the others were assigned into the group classified as “without RCA intervention.” In this study, we evaluated the endpoints as the (1) 30-day cardiovascular death, (2) 30-day total death, (3) 3-year cardiovascular death, (4) 3-year total death, (5) 3-year new myocardial infarction, and (6) 3-year right coronary artery intervention.

The SYNTAX score was used to assess the complexity of CAD. The SYNTAX score calculation was completed by two experienced cardiologists who were blinded to the procedural data and clinical outcomes. Unprotected LM CAD was defined as a left main lesion with a diameter stenosis >50% without previous PCI or CABG. A mean serum total cholesterol level >200 mg/dl without statin use at the time of this study or recorded before the present statin therapy was classified as hyperlipidemia. Acute ST-elevation myocardial infarction was defined as symptoms consistent with acute coronary syndrome and a typical rise and fall of troponin-I, with ST-segment elevation on a 12-lead electrocardiogram. Acute non-ST-elevation myocardial infarction was defined as symptoms consistent with acute coronary syndrome and a typical rise and fall of troponin-I, without ST-segment elevation on a 12-lead electrocardiogram. RCA disease was defined as “focal disease” if any lesion length <10 mm, “tubular disease” if any lesion >10 mm and <20 mm, and “diffuse disease” if any lesion >20 mm. Death from cardiovascular causes was defined as death because of acute myocardial infarction, heart failure, cardiogenic shock, ventricular arrhythmia, or cerebrovascular events. Any unidentified death was attributed to CV causes. RCA revascularization was defined as any percutaneous intervention or surgical bypass of any segment of the RCA vessel.

### 2.3. Statistical Analysis

Categorical variables were presented as counts and percentages, and the difference between the two groups was determined by the chi-square or Fisher's exact test. Continuous variables were presented as the mean ± standard deviation and were compared by Student's *t*-test, and the median value was compared using the Mann–Whitney *U* test. Survival curves were constructed with the Kaplan–Meier curves, and the log rank test with pairwise comparisons was used to calculate differences between groups. The Cox multivariate proportional hazard regression analysis was performed to investigate possible confounders. The following variables were considered in this analysis: age, sex, diabetes mellitus, hypertension, smoking, hyperlipidemia, chronic kidney disease, end stage renal disease on hemodialysis, old ischemic stroke, old MI, history of coronary artery disease, left ventricular ejection fraction (LVEF) <40%, peripheral arterial occlusive disease (PAOD), and clinical status including acute myocardial infarction, respiratory failure, ventricular tachyarrhythmia, intra-aortic balloon pump (IABP) use, temporary pacemaker use, upper gastrointestinal (UGI) bleeding, mean hospital stay, two or more stents at LM bifurcation, bare-metal stenting in LM, and intravascular ultrasound- (IVUS-) guided. The selection of variables in the multivariate model was based on a *p* value <0.1. The hazard ratio (HR) and corresponding 95% confidence intervals (CI) were reported. A *p* value <0.05 was considered significant. All data were processed using the Statistical Package for Social Sciences, version 17 (SPSS, Chicago, IL, USA), and figures were created by the GraphPad Prism 7 (GraphPad Software, Inc., La Jolla, CA).

## 3. Results

There were a total of 776 patients undergoing PCI for unprotected LM disease in our hospital between July 2001 and September 2016. In total, 235 patients with concomitant LM disease and above or equal to 70% RCA stenosis were enrolled. All patients in this study were observed for three years. There were 61 patients in the group of “without RCA intervention” and 174 patients in the group of “with RCA intervention.”

Most of these patients were male (75.7%), with hypertension (82.6%) and diabetes mellitus (60.4%). A total of 152 (64.7%) patients had chronic kidney disease or hemodialysis. There were no statistical differences between the two groups in age, body weight, sex, and comorbidities including hypertension, diabetes mellitus, hyperlipidemia, chronic kidney disease, end-stage renal disease on hemodialysis, or history of vascular events. There were 13.6% patients in the group “without RCA intervention” and 10.2% patients in the group “with RCA intervention” and had LV ejection fraction less than 40% (*p*=0.477); 24.6% patients in the group “without RCA intervention” and 32.2% patients in the group “with RCA intervention” received PCI because of acute non-ST-elevation myocardial infarction (*p*=0.266). There was no difference between the two groups in ventricular arrhythmia, respiratory failure, IABP, and temporary pacemaker use. The mean hospital stay was 13.07 ± 22.67 days (median, 6 days; interquartile range (IQR), 3–14 days) in the group “without RCA intervention” and 9.74 ± 12.63 days (median, 6 days; interquartile range (IQR), 4–9 days) in the group “with RCA intervention” (Table 1).

Table 2 showed the angiographic and procedure outcomes between the two groups. There was no significant difference of the number of diseased vessels and syntax score between the two groups. Most patients in the two groups had triple-vessel CAD (79.7% vs 84.7%, *p*=0.369). There were 44.3% patients in the group “without RCA intervention” and 50% patients in the group “with RCA intervention” and had a high syntax score >33 (*p*=0.440). There were 26.2% patients in the group “without RCA intervention” and 20.1% patients in the group “with RCA intervention” and had bare-metal stent deployed at left main lesion, and the others had drug-eluting stent deployed. Most of the patients between the two groups had IVUS-guided PCI for left main lesion (62.3% vs 72.4%, *p*=0.139) and stenting with single-stent-only strategy at the left main lesion (68.9% vs 73.6% *p*=0.479). There was no significant difference between the two stent techniques, including simultaneous kiss-stent, crushed, T-stent, and culotte's techniques. There were 36.1% patients in the group “without RCA intervention” and 47.1% patients in the group “with RCA intervention” and had only single lesion in the RCA (*p*=0.134). Others were multiple lesions, and the median lesion number was two. Only 23% patients in the group “without RCA intervention” and 15.5% patients in the group “with RCA intervention” had focal lesions only in the RCA (*p*=0.188). There were 68.9% patients in the group “without RCA intervention” and 69% patients in the group “with RCA intervention” and had diffuse lesions in the RCA (*p*=0.987). The lesion location between the two groups was similar.

**Figure 1:**
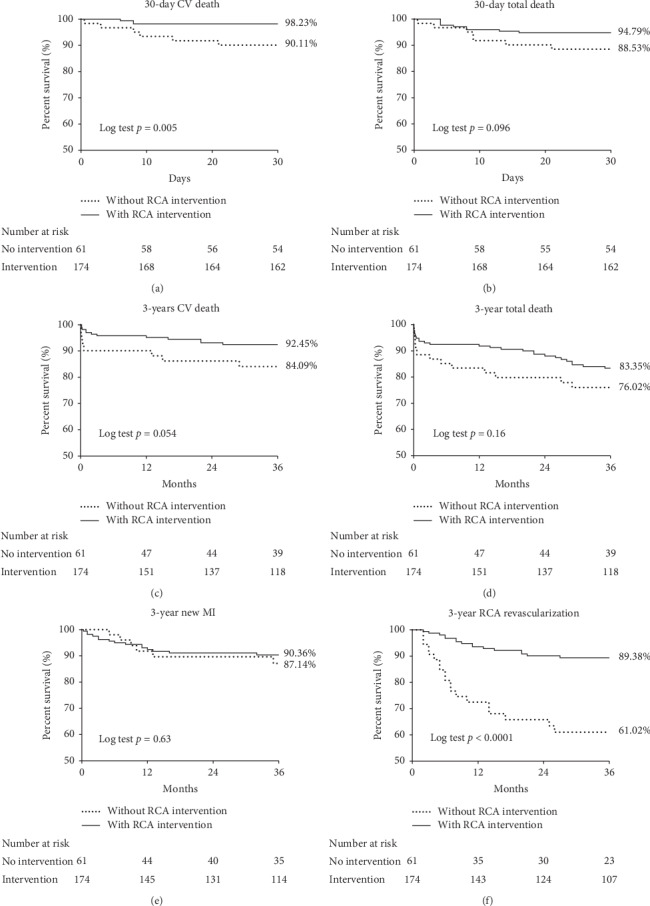
Kaplan–Meier curves for event-free survival. Solid line—patients with concomitant left main disease and RCA significant stenosis (>70% stenosis) and received PCI to left main disease and right coronary artery at the same hospitalization; dotted line—patients with concomitant left main disease and RCA significant stenosis (>70% stenosis) only received PCI to left main disease and was discharged with residual RCA significant stenosis. (a) 30-day cardiovascular death-free survival. (b) 30-day total death-free survival. (c) 3-year cardiovascular death-free survival. (d) 3-year total death-free survival. (e) 3-year new myocardial infarction-free survival. (f) 3-year RCA revascularization-free survival.

Six patients in the group “without RCA intervention” and three patients in the group “with RCA intervention” died because of cardiovascular causes within 30 days (free of the 30-day cardiovascular death; 90.11% vs. 98.23%, *p*=0.005). Seven patients in the group “without RCA intervention” and nine patients in the group “with RCA intervention” died from any cause within 30 days (free of the 30-day total death; 76.02% vs. 83.35%, *p*=0.096) (Figures 1(a) and 1(b)). Nine patients in the group “without RCA intervention” and twelve patients in the group “with RCA intervention” had cardiovascular death within three-year, and there was no statistical difference between the two groups (free of the 3-year cardiovascular death; 84.09% vs.92.45%, *p*=0.054). There was no significant difference in the 3-year all-cause death (free of the 3-year total death; 76.02% vs 83.35%, *p*=0.16), with 14 patients in the group “without RCA intervention” and 27 patients in the group “with RCA intervention” dead at the end of three years (Figures 1(c) and 1(d)). There was no increase in the 3-year new myocardial infarction rate of patients in the “without RCA intervention” group at index hospitalization (free of new myocardial infarction; 87.14% vs 90.36%, *p*=0.63) (Figure 1(e)). But patients without RCA intervention had extremely high risk to receive RCA revascularization within the next 3 years as compared to those with RCA intervention (free of the 3-year RCA revascularization; 61.02% vs 89.38%, *p* < 0.0001) (Figure 1(f)). A total of 19 patients in the group “without RCA intervention” will receive RCA PCI in the next 3 years, and 14 of them will receive PCI during the next first year.

The univariate analysis showed that “without RCA intervention,” female, IABP use, temporary pacemaker use, respiratory failure, and implanted bare-metal stent at left main lesion were the risk factors of the 30-day cardiovascular death. The multivariate analysis showed that “without RCA intervention” was still the independent risk factor for the 30-day cardiovascular death (HR = 9.37, 95% CI 1.34–65.39. *p*=0.024). Other independent risk factors include IABP and temporary pacemaker use, respiratory failure, and bare-metal stent implantation at left main lesion (Table 3).

The univariate analysis showed that without RCA intervention, body weight, chronic kidney disease, end-stage renal disease on hemodialysis, history of myocardial infarction, PAOD, and LVEF <40% were the risk factors for the 3-year RCA revascularization. But only “without RCA intervention” and end-stage renal diseases were the independent risk factors in the multivariate analysis (Table 4).

## 4. Discussion

This study provides evidence that PCI for concomitant RCA stenosis with LM intervention during the same hospitalization significantly lowers the 30-day cardiovascular death and 3-years RCA revascularization rate compared to patients who do not receive PCI for RCA stenosis. Most of the patients without RCA intervention at the index hospitalization would receive RCA intervention for any reason within 3 years. Patient without receiving RCA intervention during the same hospitalization will not increase the 30-day total death, 3-year total death, and 3-year new myocardial infarction rate.

A previous study had reported that a patient with unprotected LM disease and concomitant RCA disease (RCA stenosis more than 50%) had a worse 30-day survival rate, and the RCA disease was the independent predictor of total death [7]. Our study further demonstrated that the RCA intervention in those concomitant LM and RCA diseased patients at the index hospitalization would improve the 30-day cardiovascular death. After adjusting other risk factors, RCA intervention is still the independent risk factor of the 30-day cardiovascular death. Without RCA intervention, these patients will result in more than a nine-fold risk of 30-day cardiovascular death (HR = 9.37, 95% CI: 1.34–65.39. *p*=0.024).

Our study also showed that residual RCA stenosis had an influence on the 3-year cardiovascular death. Although our study failed to achieve the statistical difference, it showed that residual RCA stenosis had a trend of increasing the 3-year cardiovascular death. Capodanno et al. reported that a patient with residual RCA stenosis had a more than four times higher cardiac death (HR 4.41, 95% CI 1.55–12.51, *p*=0.005) at the 3-year follow-up [4]. The number of patients in our study is relatively small, and a large portion of these patients in the group of “without RCA intervention” received RCA intervention in the first year after discharge and may interfere in the outcome of the 3-year cardiovascular death in our study.

In patients with left main and multiple vessel disease, most of the patients can achieve complete revascularization after CABG intervention. But if they receive PCI to the left main disease, the interventionists may not treat the RCA stenosis at the index hospitalization. The importance of “complete revascularization” regardless of PCI or CABG in complex coronary artery disease was demonstrated by previously studies [14–18]. Zhang et al. reported a patient with unprotected left main disease who did not achieve revascularization in all diseased segments with a diameter ≧2.5 mm had higher all-cause mortality and a composite of cardiac death, myocardial infarction, and repeat revascularization [17]. A meta-analysis which included 38 publications and a total of 156,240 patients concluded that incomplete revascularization in multiple vessel disease, anatomically or functionally, increased the risk of death, myocardial infarction, and repeated revascularization. And the degree of incomplete revascularization is strongly associated to the odds ratio of mortality [18]. Our study was consistent with previous studies in the 3-year revascularization rate. Without RCA intervention at the same hospitalization will significantly increase the risk of RCA revascularization in the next 3 years (HR = 3.629, 95% CI = 1.82–7.22, *p* < 0.001). The multivariate analysis in our study demonstrated that only without RCA intervention and hemodialysis are the independent risk factors of RCA revascularization. However, our study showed that the 30-day total death, 3-year total death, and 3-year new myocardial infarction showed no difference whether patient received RCA PCI or not at the same hospitalization for LM intervention. A possible explanation of no difference in the 3-year new myocardial infarction is that there were 14 over 61 patients who received RCA intervention during the next first year, and it prevented further new cardiovascular events.

There are several limitations in this study. First, it is a retrospective study, and all data were collected from a single medical center. Second, patient with or without RCA intervention is dependent on the decision of a physician and is therefore not randomized. Although neither groups had significance in clinical presentation status and syntax score, there will still be some selection bias in this study. Third, this study had a small patient population, and many patients in the group of “without RCA intervention” received RCA revascularization during the observation period. This may interfere in the long-term outcome of our study and underestimate the risk of residual RCA critical stenosis after LM revascularization. Fourth, we excluded the patients with either chronic total occlusion or RCA lesion under 70% stenosis, so our result cannot be applied in these groups. Last, we adopted the angiographic definition of the RCA critical stenosis, and not all of patients in this study received the PCI procedure under IVUS-guide. Accordingly, some patients with physiological ischemia of RCA territory were possibly excluded in our study. Despite the limitations mentioned above, given the paucity of reports regarding the benefit of concomitant PCI for RCA lesions in patients with LMCA disease and RCA stenosis, this study still provides valuable information for clinical practice.

## 5. Conclusions

Right coronary artery intervention for concomitant left main and right coronary artery stenosis at the same hospitalization will reduce the 30-day cardiovascular death and the 3-year RCA revascularization rate compared to those without RCA intervention. Without RCA intervention is both the independent risk factor for the 30-day cardiovascular death and the 3-year RCA revascularization. It is reasonable to perform RCA intervention for those patients with concomitant left main and right coronary artery disease during the same hospitalization for left main percutaneous coronary intervention.

## Figures and Tables

**Table 1:** Baseline characteristics and clinical status between the two groups.

	Without RCA intervention *N* = 61 (%)	With RCA intervention *N* = 174 (%)	*p* value
Age (years), mean	69.39 ± 9.68	67.10 ± 10.58	0.138
Body weight (kg), mean	63.93 ± 9.85	66.17 ± 11.55	0.178
Male	46 (75.4%)	132 (75.9%)	0.943
Diabetes mellitus	42 (68.9%)	100 (57.5%)	0.118
Hypertension	46 (75.4%)	148 (85.1%)	0.088
Current smokers	10 (16.4%)	42 (24.1%)	0.210
Hyperlipidemia	35 (57.4%)	111 (63.8%)	0.374
Chronic kidney disease	40 (65.6%)	94 (54%)	0.117
Hemodialysis	5 (8.2%)	13 (7.5%)	0.787^*∗*^
Previous stroke	15 (24.6%)	24 (13.8%)	0.051
Previous myocardial infarction	14 (23.0%)	27 (15.5%)	0.188
Previous coronary artery disease	25 (41.0%)	64 (36.8%)	0.560
Peripheral artery disease	8 (13.1%)	11 (6.3%)	0.094
LVEF <40%	8 (13.6%)	17 (10.2%)	0.477
Atrial fibrillation history	1 (1.6%)	8 (4.6%)	0.453^*∗*^
Warfarin or NOAC use	0 (0%)	2 (1.1%)	1.0^*∗*^
Acute NSTEMI	15 (24.6%)	56 (32.2%)	0.266
Respiratory failure	6 (9.8%)	12 (6.9%)	0.458
Ventricular arrhythmia	3 (4.9%)	4 (2.3%)	0.380^*∗*^
IABP	10 (16.4%)	26 (14.9%)	0.787
Temporary pacemaker	1 (1.6%)	5 (2.9%)	1.000^*∗*^
UGI bleeding	4 (6.6%)	6 (3.4%)	0.290^*∗*^
Hospital stay (mean)	13.07 ± 22.67	9.74 ± 12.63	0.280
Hospital stay (median)	6 (3–14)	6 (4–9)	0.528

Data are presented as the mean ± standard deviation, *n* (%), and median (IQR). ^*∗*^Fisher's exact test. IABP = intra-aortic balloon pump; LVEF = left ventricular ejection fraction; RCA = right coronary artery; NOAC = novel oral anticoagulants; NSTEMI = non-ST-elevation myocardial infarction; and UGI = upper gastrointestinal bleeding.

**Table 2:** Angiographic and procedure outcome between the two groups.

	Without RCA intervention (*n* = 61)	With RCA intervention (*n* = 174)	*p* value
SYNTAX score values	33.96 ± 11.52	33.26 ± 9.65	0.523
≤22	7 (11.5%)	14 (8%)	0.419
23–32	27 (44.3%)	73 (42.0%)	0.754
≥33	27 (44.3%)	87 (50.0%)	0.440

Number of diseased vessels	2.80 ± 0.40	2.83 ± 0.42	0.626
LM with single vessel	0 (0%)	3 (1.8%)	0.571
LM with two vessels	12 (20.3%)	23 (13.5%)	0.223
LM with triple vessels	49 (80.3%)	148 (85.1%)	0.388

PCI procedure			
BMS at LM	16 (26.2%)	35 (20.1%)	0.319
IVUS-guided	38 (62.3%)	126 (72.4%)	0.139

Total stent number in LM	1.31 ± 0.47	1.28 ± 0.49	0.681
Single stent	42 (68.9%)	128 (73.6%)	0.479
Two stents	19 (31.1%)	43 (24.7%)	0.326
Three stents	0 (0%)	3 (1.7%)	0.570

Two stent technique			
SKS	1 (1.6%)	2 (1.1%)	1.000^*∗*^
Crushed	3 (4.9%)	4 (2.3%)	0.380^*∗*^
T-stent	6 (9.8%)	23 (13.2%)	0.489
Culottes	9 (14.8%)	17 (9.8%)	0.286

RCA vessel classification			
Multiple lesions	39 (63.9%)	92 (52.9%)	0.134
Lesion numbers (median)	2 (1–2.5)	2 (1-2)	0.055
Focal disease	14 (23.0%)	27 (15.5%)	0.188
Tubular disease	5 (8.2%)	27 (15.5%)	0.151
Diffuse disease	42 (68.9%)	120 (69.0%)	0.987

RCA lesion locations			
Primary	29 (47.5%)	94 (54.0%)	0.383
Mid	31 (50.8%)	83 (47.7%)	0.675
Distal	27 (44.3%)	71 (40.8%)	0.637

Data are presented as the mean ± standard deviation, *n* (%), and median (IQR). ^*∗*^Fisher's exact test. BMS = bare-metal stent; IVUS = intravascular ultrasound; LM = left main; PCI = percutaneous coronary intervention; RCA = right coronary artery; and SKS = simultaneous kissing-stents.

**Table 3:** Cox regression for the 30-day cardiovascular death.

30 day CV death	Univariate analysis			Multivariate analysis		
	HR	95% CI	*p*	HR	95% CI	*p*
Without RCA intervention	4.548	2.334–8.862	<0.001	9.370	1.343–65.387	0.024
Female	4.188	1.124–15.599	0.033			
IABP	12.988	3.243–52.021	<0.001	7.480	1.463–38.252	0.016
Temporary pacemaker	29.095	7.416–118.459	<0.001	28.628	3.242–252.778	0.003
Respiratory failure	6.800	1.697–27.258	0.007			
LM BMS	4.623	1.241–17.217	0.022	4.766	1.108–20.510	0.036

CI = confidence interval; CV = cardiovascular; HR = hazard ratio; IABP = intra-aortic balloon pump; LM = left main; and RCA = right coronary artery.

**Table 4:** Cox regression for the 3-year RCA revascularization.

3-year RCA revascularization	Univariate analysis			Multivariate analysis		
	HR	95% CI	*p*	HR	95% CI	*p*
Without RCA intervention	5.756	1.439–23.014	0.013	3.629	1.816–7.216	<0.001
Body weight	0.964	0.935–0.993	0.017			
Diabetes mellitus	2.026	0.973–4.222	0.059			
Chronic kidney disease	3.555	1.552–8.143	0.003			
Hemodialysis	4.706	2.037–10.872	<0.001	3.421	1.379–8.488	0.008
Previous MI	2.251	1.080–4.693	0.030			
PAOD	4.367	1.803–10.576	0.001			
LVEF <40%	3.182	1.387–7.299	0.006			

CI = confidence interval; HR = hazard ratio; IABP = intra-aortic balloon pump; LM = left main; LVEF = left ventricular ejection fraction; MI = myocardial infarction; PAOD = peripheral arterial occlusive disease; and RCA = right coronary artery.

## Data Availability

The data used to support the findings of this study are available from the corresponding author upon request.
